# Supplementary material for: Endoplasmic reticulum phospholipid scramblase activity revealed after protein reconstitution into giant unilamellar vesicles containing a photostable lipid reporter
Source: Sci Rep. 2021 Jul 13;11:14364. doi: 10.1038/s41598-021-93664-0 (PMC8277826; doi:10.1038/s41598-021-93664-0)
Supplement: Supplementary file 1 — Suppl. Fig. S1 outlines and validates liposome reconstitution with fluorescently labeled endoplasmic reticulum (ER) membrane proteins and verifies their scramblase activity after fluorescence labeling. Suppl. Fig. S2 provides additional data on eGUVs to support the results shown in Fig. 4b. [file 41598_2021_93664_MOESM1_ESM.docx]

**Endoplasmic reticulum phospholipid scramblase activity revealed after protein reconstitution into giant unilamellar vesicles containing a photostable lipid reporter**

Patricia P. M. Mathiassen^1^, Anant K. Menon^2*^ and Thomas Günther Pomorski^1,3*^

**Supplementary Information**

This file includes all supplementary information for the manuscript: Figures S1 and S2

**Supplementary figure S1**

**
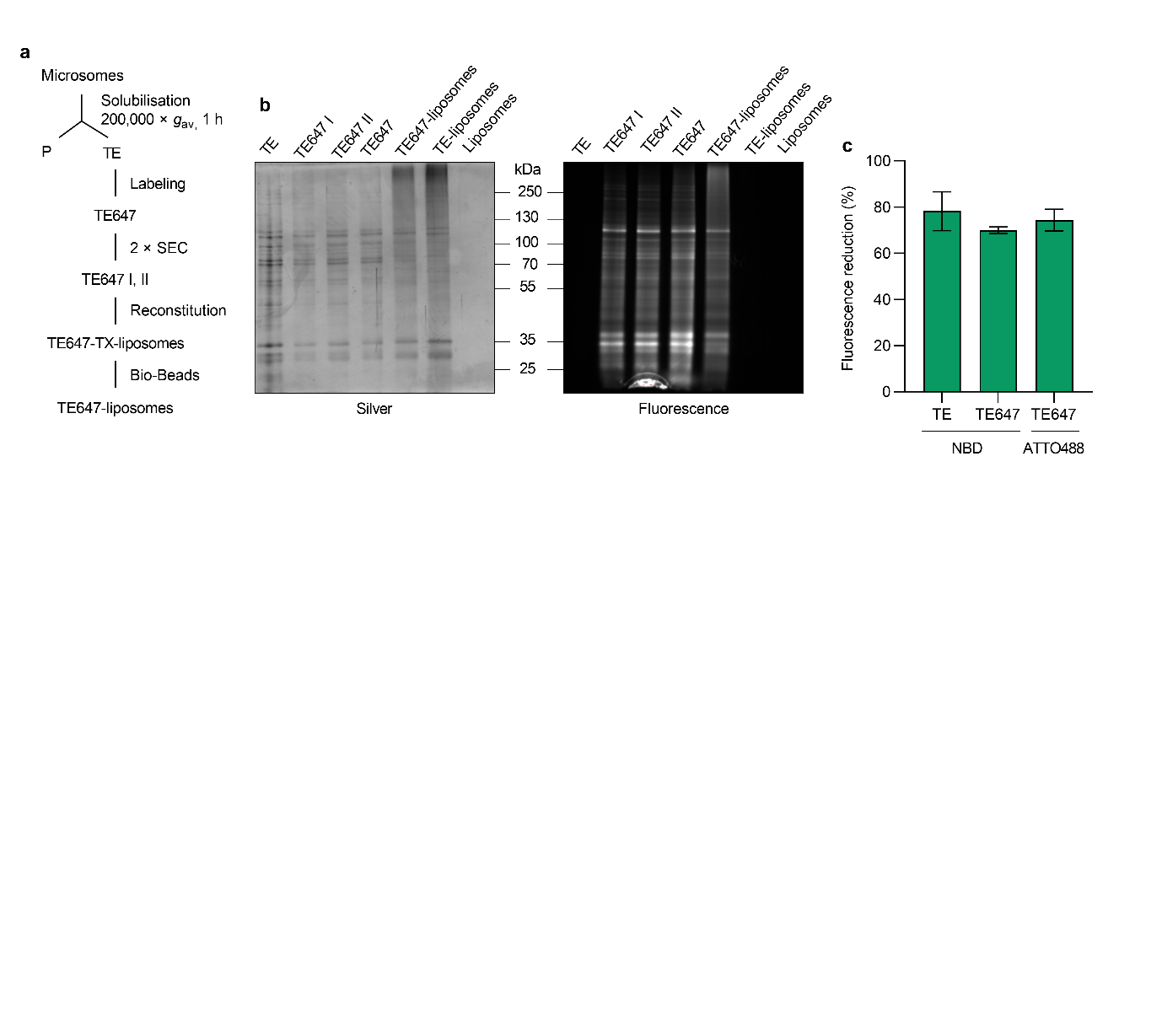
**

**Supplementary figure S1. Preparation of fluorescently labeled endoplasmic reticulum (ER) membrane proteins and reconstitution into large unilamellar liposomes.** (**a**) Experimental workflow. ER membrane proteins were selectively solubilized from yeast microsomes with Triton X-100 to generate a ‘Triton Extract’ (TE). TE was fluorescently labeled (TE647) with Alexa Fluor 647 NHS ester, excess fluorophore was removed by two rounds of size-exclusion chromatography (SEC), yielding TE647 I and subsequently TE647 II. TE647 II was reconstituted together with egg PC, ATTO488-PE, and biotin-PE into liposomes (TX-liposomes) using Bio-Beads SM-2 to remove detergent. Proteoliposomes were also generated with unlabeled TE and protein-free liposomes were generated in parallel. (**b**) SDS-PAGE analysis of samples from different steps during proteoliposome reconstitution. The gel was visualized by silver staining (left) and fluorescence scan of Alexa Fluor 647 (right). (**c**) Percentage of dithionite-mediated fluorescence reduction of C6-NBD-PC and ATTO488-PE-containing proteoliposomes (NBD and ATTO488, respectively) reconstituted with TE or TE647. Results are presented as mean ± s.d. of at least two technical replicates.

**Supplementary figure S2**

**
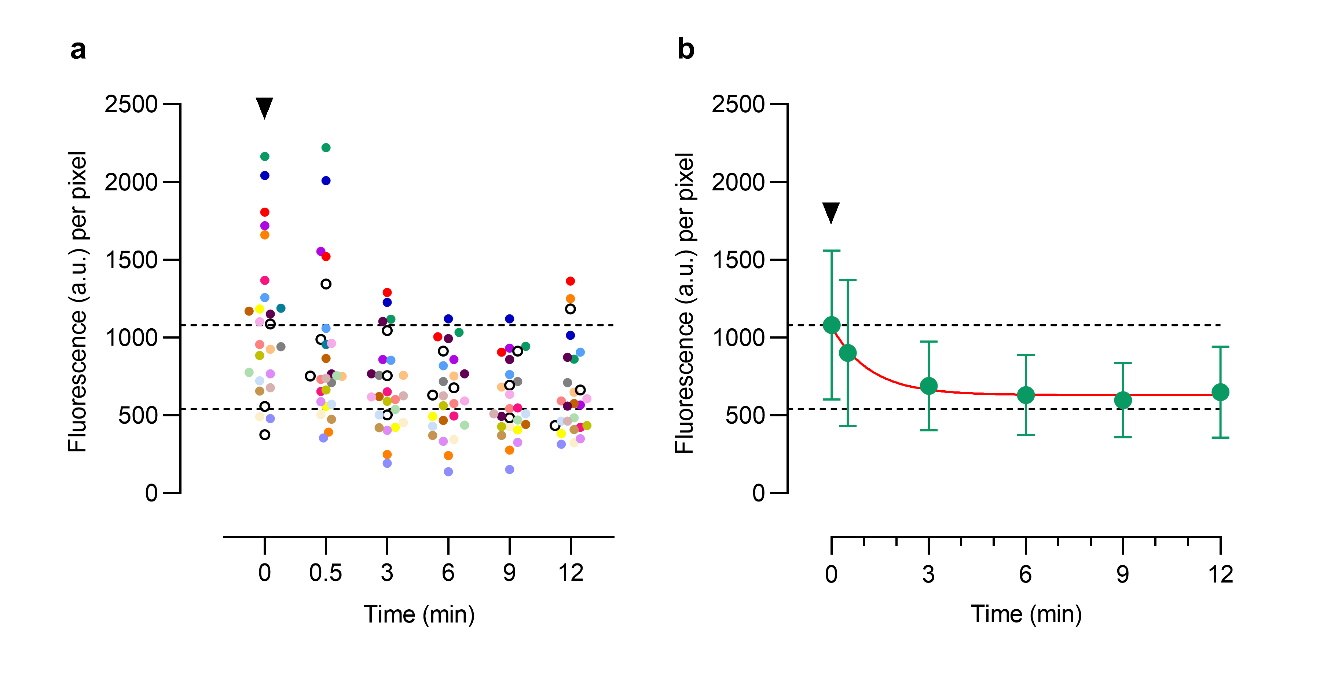
**

**Supplementary figure S2. Dithionite bleaching of ATTO488-PE in empty GUVs.** (**a**) Dot plot of ATTO488-PE fluorescence intensities of individual empty giant unilamellar vesicles (eGUVs; each eGUV is uniquely color coded, n=26) before (t=0 min) and after dithionite addition (t=0.5, 3, 6, 9, and 12 min). Black open circles indicate three eGUVs that did not react to dithionite. Dashed lines indicate 100% and 50% levels of fluorescence based on the mean value at t=0 min. (**b**) Time course of the loss of average ATTO488-PE fluorescence intensity of eGUVs upon addition of dithionite (arrowhead). Data are compiled from panel a and presented as mean ± s.d. (n=26). The red line represents a monoexponential fit of the data (t_½_ = 0.82 min). Dashed lines indicate 100% and 50% levels of fluorescence based on the mean value at t=0 min.
